# Traditional Chinese health-preserving exercises and self-worth in older adults: a moderated mediation model

**DOI:** 10.3389/fpsyg.2026.1771130

**Published:** 2026-10-06

**Authors:** Doudou Yang, Huijun Han, Qiong Yao

**Affiliations:** 1 Department of Physical Education, Guangdong University of Science and Technology, Dongguan, China; 2 School of Sports Technology, Guangzhou College of Applied Science and Technology, Zhaoqing, China; 3 School of Wushu, Guangzhou Sport University, Guangzhou, China

**Keywords:** older adults, self-identity, self-worth, social support, traditional Chinese health-preserving exercises

## Abstract

**Objective:**

This study examined the association between traditional Chinese health-preserving exercises and self-worth in older adults, focusing on the mediating role of self-identity and the moderating role of social support.

**Method:**

A cluster sampling approach was adopted to recruit 500 older adults from 12 representative Tai Chi and Health Qigong associations in the Guangdong–Hong Kong–Macao Greater Bay Area for a cross-sectional survey. Data were collected using the Traditional Chinese Health-Preserving Exercise Scale, the Self-Identity Scale, the Social Support Scale, and the Self-Worth Scale. Amos 26.0 was used to assess the reliability and validity of the scales. SPSS 27.0 was employed to conduct descriptive statistics and correlation analyses for the four variables, followed by multiple linear regression to examine the total association. Subsequently, the SPSS PROCESS macro (Models 4 and 7) was used to test the mediating role of self-identity and the moderated mediation model, respectively.

**Results:**

Significant positive correlations were observed between any two of the four variables: traditional Chinese health-preserving exercises, self-identity, social support, and self-worth (*p* < 0.01). Multiple linear regression analysis showed that traditional Chinese health-preserving exercises were significantly and positively associated with self-worth (*β* = 0.104, *p* < 0.05). The indirect pathway from traditional Chinese health-preserving exercises to self-worth through self-identity was significant [*β* = 0.095, Bootstrap 95% CI (0.018, 0.165)]. Moreover, social support moderated the association between traditional Chinese health-preserving exercises and self-identity, which was strongest at high levels of social support (*β* = 0.365, *p* < 0.001), and the mediating role of self-identity was significant only at high levels of social support [*β* = 0.143, Bootstrap 95% CI (0.052, 0.231)].

**Conclusion:**

Drawing on the theoretical frameworks of self-worth, self-identity, and social support, this study suggests that among older adults who enjoy robust social support networks, regular engagement in traditional Chinese health-preserving exercises is associated with the development and reinforcement of self-identity across physiological, psychological, and social dimensions. Within this process, older adults may become increasingly oriented toward contemplating the meaning of life in a natural context, shifting from a focus on external achievements toward exploring inner peace and the inherent value of life, and this reorientation may accompany the fulfillment of self-worth.

## 1 Introduction

Global population aging is accelerating, presenting substantial challenges to population structure and labor supply worldwide. According to the 2025 World Population Situation Report released by the United Nations Population Fund (UNFPA), 55% of countries and regions globally now have a total fertility rate below the replacement level of 2.1, with 63 countries and regions reporting rates below 1.5 (Khaleghi et al., 2025). It is projected that by 2050, the global population aged 65 and over will double, reaching 1.6 billion. China’s population age structure is undergoing a similarly profound transformation. Data from the National Bureau of Statistics of China indicate that by the end of 2024, the population aged 60 and over had surpassed 300 million for the first time, and it is estimated that by 2032, individuals aged 65 and over will account for more than 20% of the total population, posing significant challenges to Chinese socioeconomic development (Chan et al., 2024).

Addressing population aging has emerged as a critical 21st-century challenge for global public health. Following the World Health Organization’s introduction of the active aging framework, scholarly attention has shifted from merely extending lifespan to realizing both personal and societal value through its three pillars: health, social participation, and security (World Health Organization, 2021). Accordingly, the aspirations of older adults are evolving from basic survival toward personal development, with increasing emphasis placed on self-worth and quality of life, both central to health and active aging.

Physical exercise is well established as an effective means of promoting older adults’ physical and mental health, contributing to enhanced self-worth and quality of life (Ghenta et al., 2022). However, although the general benefits of physical exercise for older adults’ psychological well-being are well documented, three notable gaps remain. First, most studies have treated physical exercise as an undifferentiated category and have focused predominantly on general forms of physical activity such as running and aerobic training; comparatively little attention has been paid to how different types of exercise, each carrying distinct physical, psychological, and social properties, may differentially shape psychological outcomes. Second, research on how exercise is associated with psychological well-being in older adults has focused largely on physiological pathways such as cardiovascular fitness and inflammatory markers, while psychological pathways, including self-identity formation and meaning-making, remain underexplored. Third, the evidence base draws predominantly on Western populations, with scarce attention to culturally embedded, non-Western forms of exercise and their distinct pathways.

Traditional Chinese health-preserving exercises are a form of ethnic traditional sport originating in China. Refined over millennia, they are guided by the philosophical principle of “harmony between humans and nature” and integrated with traditional Chinese medical theories, including the doctrines of Yin-Yang and the Five Elements (Jiang and Zou, 2013). Through sustained historical practice, they have evolved into a distinctive system of health-preserving thought and technique. Compared with general sports, traditional Chinese health-preserving exercises exhibit three defining features. First, mind–body unity: whereas general sports center on physical movement, traditional Chinese health-preserving exercises require the coordinated engagement of movement, breath regulation, and mental focus, constituting a holistic mode of mind–body training. Second, cultural foundation: whereas general sports are typically grounded in modern exercise science, traditional Chinese health-preserving exercises are rooted in the Daoist philosophy of “harmony between humans and nature” and the Yin-Yang and Five Elements theories of traditional Chinese medicine, carrying a distinct cultural significance. Third, practice mode: group activities in general sports typically revolve around competition or cooperation, as in ball games, whereas traditional Chinese health-preserving exercises such as Tai Chi and Ba Duan Jin, although commonly performed in groups, require neither confrontation nor coordination; each participant moves at their own pace. This model of shared practice without interdependence preserves the social atmosphere of collective participation while eliminating competitive pressure, making it especially suited to the physical and psychological characteristics of older adults (Woo, 2024).

As a culturally embedded form of exercise with distinctive mind–body and social properties, traditional Chinese health-preserving exercises offer a well-suited context for addressing these three gaps. Previous research has documented their value for health promotion among older adults; however, a systematic review of the literature reveals that existing studies have focused primarily on overall health indicators, and discussions of the psychological dimension have remained broad in scope, stopping short of examining the underlying pathways in depth. In this study, self-worth denotes an individual’s overall evaluation of their value as a person. Although this global, evaluative dimension of the self-concept has historically been labeled self-esteem, the present study consistently refers to it as self-worth. Self-worth is distinguished here from self-identity, which concerns the cognitive content of the self, and from broader psychological well-being, which encompasses affective and functional aspects of adjustment. The present study therefore conducted a cross-sectional survey of older adults across the Guangdong-Hong Kong-Macao Greater Bay Area. Drawing on self-worth theory, self-identity theory, and social support theory, this study investigates the associations among traditional Chinese health-preserving exercises, self-identity, self-worth, and social support in older adults, to provide a scientific reference for the implementation of global active aging strategies.

## 2 Theoretical research hypotheses

### 2.1 Traditional Chinese health-preserving exercises and self-worth

Self-worth is regarded as a positive psychological resource in the field of psychology, and its level significantly influences the physical and mental health of older adults. It has been defined both objectively as the ratio of achievements to aspirations, that is, “Self-worth = Achievement/Aspiration” (Bounds et al., 2024), and subjectively as a judgment of “whether I am valuable” arising from one’s own assessment and the perceived evaluations of others (Rogers, 1957). Among older adults, higher self-worth is often associated with more positive affect, reflecting better mental health and higher quality of life. Enhancing self-worth among older adults therefore constitutes a crucial pathway toward promoting active aging (Hsu and Lu, 2018).

Older adults are encouraged to increase their participation in physical exercise. The social interaction, health-promoting, health-preserving, and humanistic educational functions of sports activities may foster a greater sense of self-worth. Recent research indicates that adopting health-promoting behaviors such as physical exercise and possessing self-worth are effective means of maintaining health among older adults (Zarghami et al., 2025). Collectively, such findings suggest a positive association between physical exercise participation and self-worth among older adults.

According to the theory of self-worth, the highest level of an individual’s self-worth stems from a fundamental affirmation of the purpose and value of one’s own existence, rather than relying solely on external achievements or social comparison (Zarghami et al., 2025). Traditional Chinese health-preserving exercises, grounded in the philosophical foundation of “harmony between humans and nature” and the practical characteristic of “mind–body unity,” offer older adults a means of reorienting their self-worth. At the individual level, these exercises emphasize the synchronization of body adjustment, breath regulation, and mental regulation, requiring practitioners to perform slow, gentle, and attentive movements in coordination with breathing and consciousness. Through sustained practice, traditional Chinese health-preserving exercises impart to older adults a way of nurturing life and regulating the spirit, encouraging them to contemplate the meaning of life within the natural world. This process is associated with a shift away from concerns about linear physical decline and toward a reframing of later life as a stage of cultivating well-being, a phase devoted to pursuing inner harmony, spiritual fulfillment, and the transmission of traditional culture. Such deepened awareness and philosophical elevation regarding the meaning of life are linked to the consolidation and affirmation of self-worth among older adults. Therefore, we propose that participation in traditional Chinese health-preserving exercises is associated with self-worth among older adults.

### 2.2 The mediating role of self-identity

Self-identity, as a pivotal concept in psychology, was introduced by the American psychologist Erik H. Erikson within his theory of psychosocial development stages. It primarily referred to a coherent and stable understanding of “who I am”, which is formed through the internal integration of an individual’s personal experiences, social roles, values, and sense of group belonging. This concept addresses the fundamental question of existential “identity” (Polacsek et al., 2022). Self-identity and self-worth are two interrelated and reciprocally linked core dimensions within the self-concept, jointly reflecting an individual’s level of psychological adaptation. Scholars have delineated the relationship between them, positing that a stable and positive self-identity can enhance an individual’s level of self-worth. Conversely, self-worth is not passively determined by self-identity; it can actively contribute to the construction and maintenance of self-identity (Lundh and Foster, 2024).

As older adults confront circumstances such as retirement, bereavement, and illness, they are susceptible to negative emotions including loneliness, loss, anxiety, and depression, which can precipitate an identity crisis. The body serves as a crucial medium for the construction of self-identity in later years. The development of self-identity is closely intertwined with bodily development, which in turn is fundamentally shaped by physical exercise. Physical exercise therefore serves as a vital social resource for addressing the identity crisis among older adults. Previous research has indicated a reciprocal relationship between physical exercise and self-identity. Physical exercise engagement not only positively relates to the formation of self-identity, but the outcomes of such engagement are also associated with the individual’s level of self-identity, thereby linked to self-worth (Strachan et al., 2010).

The theory of self-identity posits that the individual is not merely a physical entity, but encompasses one’s self-perception, sense of value, and role identity (Jones and Huddlestone, 2025). As a traditional physical practice with a long history and deep cultural roots, traditional Chinese health-preserving exercises offer older adults opportunities to explore self-awareness, develop self-identity, and clarify self-positioning through practical engagement. More specifically, the practice of traditional Chinese health-preserving exercises (e.g., Tai Chi and Ba Duan Jin) allows older adults to cultivate heightened somatic awareness and to engage in the intentional modulation of their intrinsic vital energy. This physical agency may shift their initial identification away from the fixed notion of “aging as decline” (de Bruijn and van den Putte, 2012). More importantly, through sustained participation, they undergo a role transformation, gradually becoming “promoters of national fitness”, “transmitters of traditional culture”, “pursuers of mind-body unity”, and “bearers of health-preserving wisdom”. These creative and value-laden roles are internalized into the core of their self-concept through embodied experience (Conde-Pipó et al., 2021). Such reignition of vital energy, opening the possibility for the reproduction and re-creation of life, signals a transformation and sublimation of self-identity among older adults. It is within this renewed self-identity that older adults perceive their internal growth and social value, which is associated with stronger self-worth. Therefore, we hypothesize that traditional Chinese health-preserving exercises is indirectly associated with self-worth among older adults through the creative transformation of self-identity.

### 2.3 The moderating role of social support

Social support refers to the tangible assistance or emotional support individuals obtain from others or groups when confronting difficulties (Taylor, 2011), as well as the diverse resources acquired from one’s social network to meet both physiological and psychological needs (House et al., 1988). Social support has been widely applied across various academic disciplines. Within the field of sports and health science, it promotes the physical and mental well-being of individuals. Scholars have identified a correlation between physical exercise and social support (Ewald, 2016). When older adults receive greater social support, they are able to expand their support networks, participate more actively in exercise, and experience the social functions of exercise more fully.

Further research indicates that social support plays a moderating role between physical exercise and mental health (Heller et al., 2025). High levels of support from family, friends, or the community are often associated with positive psychological traits. By facilitating engagement in exercise, such support helps older adults achieve emotional communication during group activities, thereby enhancing their self-identity. This view is corroborated by Sheehy et al. (2016), whose study of older patients with Parkinson’s disease found that, through interaction and mutual assistance in group exercise, participants obtained support and recognition, which significantly strengthened their self-identity and fostered feelings of security and self-worth.

The theory of social support elucidates how resources exchanged within social relationships operate during stress and coping processes, thereby enhancing individuals’ physical and mental health (Wallston et al., 1983). Specifically, older adults in high-support environments receive multifaceted assistance: family may provide emotional encouragement and time, which is associated with sustained participation; a harmonious atmosphere of mutual aid often characterizes group exercise, and this atmosphere is linked to stronger socio-emotional bonds; and community networks may assign new social roles accompanied by public recognition. This multifaceted support plays a crucial moderating role. It continually affirms the authenticity and perceived value of the social identity older adults experience through traditional Chinese health-preserving exercises. Under these conditions, older adults may internalize these roles more firmly and confidently, which is associated with a richer self-identity and higher self-worth. Conversely, in low-support contexts, older adults who attempt to transcend age-related constraints and pursue the social roles associated with these exercises may encounter doubt and rejection from their family, friends, or community. This can be associated with a weakened self-identity, with older adults positioned as passive recipients and linked to a lower sense of self-worth.

Therefore, social support is not an isolated factor but a critical link connecting individuals’ internal psychological transitions. It moderates the psychosocial process through which physical practice relates to identity formation, thereby constituting a situational condition relevant to the development of self-worth in older adults. We hypothesize that, relative to low social support, a high level of social support is associated with a stronger relationship between older adults’ engagement in traditional Chinese health-preserving exercises and their self-worth, mediated by an enriched self-identity.

### 2.4 The present study

In summary, the three theories together form a progressively layered theoretical framework for the present study. Self-worth theory, grounded in the philosophical foundation of “body” and the practical characteristic of “mind–body unity” inherent in traditional Chinese health-preserving exercises, elucidates the distinctive association of this form of exercise with self-worth among older adults, thereby establishing the logical starting point of this investigation. Building on this foundation, self-identity theory reveals a core mediating pathway: traditional Chinese health-preserving exercises requires the coordinated integration of movement, breath regulation, and mental focus; through continuous somatic awareness cultivated in this process, older adults may move beyond the entrenched perception of “aging as decline”. Via role transformations into “transmitters of traditional culture” and “pursuers of mind-body unity”, a creative transformation of self-identity becomes possible, and this renewed self-identity is associated with higher levels of self-worth, thus linking exercise behavior with psychological benefit. Social support theory further specifies the conditions under which this pathway operates: although traditional Chinese health-preserving exercises are practiced in a group-based, shared-participation format that offers a natural field for interpersonal interaction, whether identity development can be internalized depends on the positive feedback and role affirmation received from family, peers, and the community. In the absence of such supportive environments, physical practice alone may not readily crystallize into a stable self-identity. Together, the three theories form a coherent logical chain: self-worth theory positions the research problem, self-identity theory delineates the mediating pathway, and social support theory identifies the boundary condition.

Grounded in this framework, the present study constructs a moderated mediation model (see Figure 1) with traditional Chinese health-preserving exercises as the independent variable, self-identity as the mediating variable, social support as the moderating variable, and self-worth as the dependent variable. This model aims to examine the pathways through which traditional Chinese health-preserving exercises are associated with self-worth among older adults, to identify potential heterogeneity within these pathways, and to offer preliminary practical implications for the physical and mental well-being of the older adult population. The following hypotheses are proposed.

**Figure 1:**
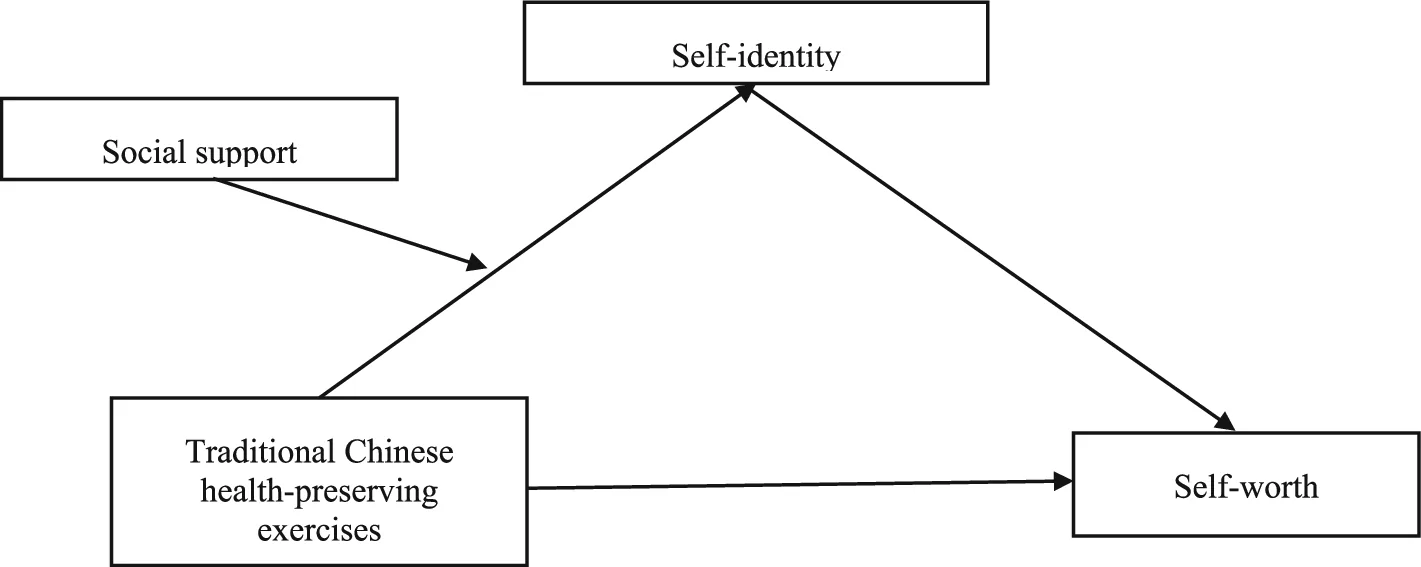
Moderated mediation model.

H1: Participation in traditional Chinese health-preserving exercises is positively associated with self-worth among older adults.

H2: Self-identity mediates the association between traditional Chinese health-preserving exercises and self-worth among older adults.

H3: Social support positively moderates the association between traditional Chinese health-preserving exercises and self-identity among older adults, such that this association is stronger at higher levels of social support.

## 3 Materials and methods

### 3.1 Participants

This study aimed to examine the association between traditional Chinese health-preserving exercises and self-worth among older adults in the Guangdong–Hong Kong–Macao Greater Bay Area, thereby informing the inheritance and development of these practices and the improvement of older adults’ mental health. Through field surveys, representative Tai Chi associations and Health Qigong associations were selected from four cities in Guangdong Province (Guangzhou, Shenzhen, Foshan, and Dongguan), as well as from the two special administrative regions of Hong Kong and Macao, yielding a total of 12 associations. These associations were professional, non-profit social organizations voluntarily formed by local Tai Chi and Health Qigong enthusiasts, with the purpose of promoting traditional Chinese health-preserving culture and preserving the development of these practices. The twelve associations were selected not only for their representative socioeconomic backgrounds but also because they regularly organized older adults to participate in Tai Chi and Health Qigong competitions, reflecting the outcomes of their sustained practice, and had accumulated numerous honors, and had established strong credibility in their respective localities. The participants were registered members of these 12 associations. Using cluster sampling, questionnaires were distributed to 550 eligible participants.

The inclusion criteria for the older adults in this study were as follows: (1) Age: 60–80 years. This range was selected because 60 years is the conventional threshold of older adulthood, whereas pronounced functional decline above 80 years may compromise safe and effective participation. Within this range, physical functions including mobility, muscle strength, organ function, motor coordination, perceptual abilities, and nervous and immune system functioning remain relatively intact, generally allowing participation in slow, gentle physical activities. (2) Health status: no cognitive or mental disorders, and no severe visual or hearing impairment (health reports could be provided by the respective associations). (3) Consistent engagement in traditional Chinese health-preserving exercises and voluntary participation in this study. Exclusion criteria included conditions that hindered communication or questionnaire completion, as well as incomplete questionnaire responses.

### 3.2 Procedure

First, the Ethics Review Committee of Guangdong University of Science and Technology approved this study. All procedures complied with the ethical standards outlined in the 1964 Declaration of Helsinki and its subsequent amendments. Second, participants were fully informed of the research purpose, which was solely for scientific inquiry. Voluntary participation was emphasized, and participants were advised of their right to withdraw at any time without consequence. Third, participants were asked about their long-term engagement and personal reflections on traditional Chinese health-preserving exercises. Based on their practice experience and physical and psychological condition, they responded to questions regarding self-identity, social support, and self-worth, and provided basic demographic information. Then, participants with normal literacy signed the informed consent form themselves. For those with limited literacy or difficulty writing, the participants first personally confirmed their consent, after which a family member signed the form on their behalf as a witness to support the participants’ decisions. Fourth, this study received strong support from the local Tai Chi and Health Qigong associations and from participants’ family members. The association leaders assisted in distributing the electronic questionnaires via the Wen Juan Xing online platform. Fifth, under the supervision of the association leaders, the researchers patiently guided the participants through the questionnaire, all of whom ultimately completed it anonymously. Finally, as a token of appreciation, all participants received a Tai Chi-themed bookmark.

This study distributed questionnaires to 550 older adults across 12 Tai Chi and Health Qigong associations in the Guangdong–Hong Kong–Macao Greater Bay Area. Controlling for demographic variables including gender, age, practice format, educational background, economic income, marital status, number of children, living arrangement, and physical condition, the study examined the association between traditional Chinese health-preserving exercises and self-worth among older adults, as well as the moderating role of social support in the mediating pathway of self-identity. Questionnaires that were carelessly completed or in which a single response option was selected across all items were treated as invalid and excluded. After questionnaire screening, 500 valid questionnaires were retained. The removal of 50 invalid questionnaires was necessary because indiscriminate or uniform response patterns suggested that the respondents had difficulty comprehending the items, reflecting a casual or perfunctory approach to participation. More importantly, these invalid data could not faithfully capture the associations among traditional Chinese health-preserving exercises, self-identity, self-worth, and social support. Their inclusion would have distorted the estimation of the main association, mediation, and moderated mediation analyses, thereby raising concerns about the credibility of the study’s conclusions.

In total, 500 valid questionnaires were returned, yielding a valid response rate of 91%. The sample comprised 500 older adults (232 males, 268 females). Regarding educational attainment, 169 participants held a bachelor’s degree or higher, and 331 had completed education below the bachelor’s level. With respect to living arrangements, 81 participants lived alone and 419 did not. The distribution of marital status was as follows: 431 married, 39 single, 9 divorced, and 21 widowed. In terms of practice format, 244 participants engaged in individual practice and 256 in group practice.

### 3.3 Measures

#### 3.3.1 The scale of traditional Chinese health-preserving exercises

The traditional Chinese health-preserving exercises scale employed in this study was adapted from the Physical Activity Rating Scale (PARS-3; Liang, 1994) and modified to reflect both the characteristics of older adults engaged in traditional Chinese health-preserving exercises and the specific aims of the present research. The scale consists of three items (e.g., “How long is each of your traditional Chinese health-preserving exercise sessions?” and “How frequently and at what intensity do you practice traditional Chinese health-preserving exercise?”), assessing the duration, frequency, and intensity of practice, respectively. Exercise volume is calculated as the product of these three dimensions (score range: 0–100) and classified into low (≤19), moderate (20–42), and high (≥43) levels. Because the total score is derived from three dimensions that jointly define the construct of exercise volume rather than serving as its outward manifestations, the scale is properly treated as a formative indicator. Consistent with this conceptualization, we evaluated the measurement quality of the scale through three lines of evidence rather than through methods grounded in a reflective framework (e.g., confirmatory factor analysis and composite reliability): (1) test–retest reliability: read ministration to the same group of older adults after a two-week interval yielded a correlation of *r* = 0.87, indicating good temporal stability; (2) criterion validity: using the PARS-3 as the criterion, the criterion-related validity coefficient was *r* = 0.82; and (3) content validity: six domain experts from Guangzhou Sport University independently rated each item’s relevance to the exercise volume construct on a 4-point scale, producing an item-level content validity index (I-CVI) of 1.00 for all three items and a scale-level index (S-CVI/Ave) of 1.00, well above the recommended thresholds (I-CVI ≥ 0.83, S-CVI/Ave ≥ 0.90), confirming that all items adequately represent the target construct.

#### 3.3.2 The scale of self-identity

This study adopted the Self-identity Scale developed by Ochse and Plug (1986) to measure the level of self-identity in older adults. The scale is unidimensional and therefore requires no second-order factor modeling. It consists of 19 items, of which 7 are reverse-scored and 12 are positively scored. Responses are recorded on a 5-point Likert scale ranging from 1 (“strongly disagree”) to 5 (“strongly agree”), with higher total scores reflecting stronger self-identity. Confirmatory factor analysis indicated excellent model fit: *χ*^2^/df = 2.686, RMSEA = 0.058, SRMR = 0.012, CFI = 0.995, IFI = 0.995.

#### 3.3.3 The scale of social support

This study employed the Social Support Scale developed by Cohen and Matthews (1987), which assesses individuals’ perceived social support. The scale is multidimensional, consisting of 12 items that measure family support, friend support, and other support, with 4 items per dimension. Responses are recorded on a 5-point Likert scale ranging from 1 (“strongly disagree”) to 5 (“strongly agree”). All items are positively scored, with higher total scores reflecting greater subjective social support. A second-order confirmatory factor analysis indicated excellent model fit: *χ*^2^/df = 2.833, RMSEA = 0.017, SRMR = 0.032, CFI = 0.972, IFI = 0.972.

#### 3.3.4 The scale of self-worth

This study measured self-worth using the Rosenberg Self-Esteem Scale (Rosenberg, 1965), one of the most widely recognized instruments for assessing self-worth and self-esteem. Although conventionally labeled a self-esteem scale, the instrument measures individuals’ overall evaluation of their worth as a person, which is the global, evaluative construct termed self-worth in the present study; it is therefore referred to as the self-worth scale throughout this manuscript. Originally designed to measure adolescents’ overall feelings of self-worth and self-acceptance, the scale has since been extensively applied across diverse age groups and cultural contexts, particularly among older adult populations. The scale is unidimensional and consists of 10 items, of which 4 are reverse-scored and the remaining 6 are positively scored. Responses are recorded on a 5-point Likert scale ranging from 1 (“strongly disagree”) to 5 (“strongly agree”), with higher total scores reflecting greater self-worth. Confirmatory factor analysis indicated excellent model fit: *χ*^2^/df = 1.447, RMSEA = 0.010, SRMR = 0.004, CFI = 0.995, IFI = 0.995.

### 3.4 Data analysis

SPSS 27.0 and Amos 26.0 were used to evaluate the reliability and validity of the data. Scale reliability was assessed using Cronbach’s alpha coefficients and composite reliability (CR). The Cronbach’s alpha values for self-identity (0.918), social support (0.768), and self-worth (0.919) all exceeded 0.70, indicating satisfactory internal consistency. Measurement validity was evaluated through confirmatory factor analysis (CFA) in Amos 26.0. The overall model fit indices (*χ*^2^/df = 2.286, CFI = 0.980, NFI = 0.965, IFI = 0.980, RMSEA = 0.051, SRMR = 0.035) indicated a good fit between the measurement model and the data.

The composite reliability (CR) values for self-identity (0.919), social support (0.769), and self-worth (0.920) all exceeded the 0.70 threshold, further supporting the internal consistency of the measures. The average variance extracted (AVE) values for self-identity (0.695) and self-worth (0.741) exceeded the 0.50 benchmark, indicating strong convergent validity. Although the AVE for social support (0.455) fell slightly below this threshold, its composite reliability of 0.769 and satisfactory model fit provided only partial support for convergent validity, and this scale should therefore be interpreted with some caution. Discriminant validity was assessed via the latent-variable correlation matrix estimated in confirmatory factor analysis (see Table 1). The square roots of the AVE for each construct exceeded the corresponding latent correlations, supporting satisfactory discriminant validity. Overall, the measurement model exhibited satisfactory reliability and validity, providing an adequate basis for testing the research hypotheses.

**Table 1:** Discriminant validity of the measures and latent-variable correlation matrix.

	SI	SS	SW
SI	**0.833**		
SS	0.441	**0.674**	
SW	0.774	0.413	**0.860**

The numbers on the diagonal are the square root of the average variance extracted (AVE). Off-diagonal values are latent-variable correlations estimated in confirmatory factor analysis. SI, self-identity; SS, social support; SW, self-worth.

Accordingly, the following analyses were performed in sequence. First, Harman’s single-factor test was conducted using SPSS 27.0 to provide a preliminary examination of common method bias. Second, descriptive statistics were computed to describe the basic characteristics of each variable, while Pearson correlations among the observed variables were used to examine the linear relationships among them. Third, linear regression analysis was used to assess the association between traditional Chinese health-preserving exercises and self-worth among older adults. Fourth, Models 4 and 7 of the PROCESS macro were employed to analyze the mediating role of self-identity and to test the moderated mediation of social support, respectively. Finally, because the sample was nested within 12 associations, intraclass correlation coefficients (ICCs) of the core variables were calculated to assess potential clustering effects, and robustness checks were performed using cluster-robust standard errors clustered at the association level.

## 4 Results

### 4.1 Common method deviation test

Because the data in this study were obtained from older adults’ self-reports, Harman’s single-factor test was employed to assess common method bias. The results indicated the presence of three factors with eigenvalues greater than 1, with the first factor accounting for 38.13% of the total variance, which fell below the critical threshold of 40%. This finding provides preliminary evidence that common method bias is not a serious concern. However, given the inherent limitations of Harman’s single-factor test, this evidence should be regarded only as a limited diagnostic and cannot fully rule out the presence of common method bias.

### 4.2 Descriptive statistics and correlation analysis

Preliminary analyses of the four study variables included descriptive statistics for each variable and Pearson correlations among the observed variables (see Table 2). Traditional Chinese health-preserving exercises were positively and significantly correlated with self-identity, social support, and self-worth (*p* < 0.01). On the basis of these correlational findings, a sequential analytical framework was adopted: the total association was examined first, followed by the mediation analysis, and finally the moderated mediation model.

**Table 2:** Descriptive statistics and observed-variable correlations coefficients (*N* = 500).

Variables	TCHPE	SI	SS	SW
TCHPE	1			
SI	0.167^**^	1		
SS	0.259^**^	0.441^**^	1	
SW	0.141^**^	0.755^**^	0.437^**^	1
M	34.74	3.991	4.259	4.123
SD	21.65	0.863	0.564	0.962

Significance level: ^**^p < 0.01. TCHPE, traditional Chinese health-preserving exercises; SI, self-identity; SS, social support; SW, self-worth.

### 4.3 Test of the total association

Traditional Chinese health-preserving exercises served as the independent variable and self-worth as the dependent variable. Gender, age, practice format, education background, economic income, marital status, number of children, living arrangement, and physical condition were included as control variables. The control variables were coded as follows. Gender (1 = male, 2 = female), practice format (1 = individual, 2 = group), and living arrangement (1 = living alone, 2 = not living alone) were entered as binary variables. Age (1 = 60–64, 2 = 65–69, 3 = 70–74, 4 = 75–80 years), education background (1 = no schooling, 2 = primary school, 3 = junior high school, 4 = senior high school, 5 = associate degree, 6 = bachelor’s degree or above), economic income (1 = below 3,000, 2 = 3,000–5,000, 3 = 5,000–7,000, 4 = above 7,000 RMB), number of children (1 = none, 2 = one, 3 = two, 4 = three or more), and physical condition (1 = poor, 2 = fair, 3 = good, 4 = excellent) were treated as ordinal scores.

Marital status (1 = widowed, 2 = divorced, 3 = unmarried, 4 = married) was treated as an ordinal score, with higher scores reflecting greater partner support. This choice was based on the rationale that different marital statuses differ in their degree of partner support: married individuals have a spouse and therefore receive the greatest partner support, whereas widowed individuals have lost their spouse and receive the least. However, because marital status is inherently a nominal variable, treating it as an ordinal score may impose an artificial linear ordering and equal distance across its categories. To address this concern, we conducted a sensitivity analysis in which marital status was re-coded as dummy variables, with married as the reference category and separate dummy variables for unmarried, divorced, and widowed. The results showed that the core conclusions remained unchanged, indicating that the findings were not sensitive to the coding of marital status. Detailed results are reported in Section 4.6.

Linear regression analysis was employed to examine the total association between traditional Chinese health-preserving exercises and self-worth among older adults. The regression model was specified as follows:

$$\begin{array}{l} \text{Self Worth}=\beta_{0}+\beta_{1}\text{Practice format}+\beta_{2}\text{Gender}\\ +\beta_{3}\mathrm{Age}+\beta_{4}\text{Education background}+\beta_{5}\text{Economic income}\\ +\beta_{6}\text{Marital status}+\beta_{7}\text{Number of children}\\ +\beta_{8}\text{Living arrangement}+\beta_{9}\text{Physical condition}+\varepsilon_{i} \end{array}$$

Prior to the main analyses, all regression assumptions were examined. Scatterplots of each predictor against the outcome indicated approximately linear relationships. Standardized residuals approximated a normal distribution (skewness = −1.36, kurtosis = 1.40). A scatterplot of standardized residuals against predicted values displayed a random pattern, consistent with homoscedasticity. All variance inflation factors (VIFs) were below 2.0, ruling out multicollinearity concerns. The maximum Cook’s distance was 0.065, well below the threshold of 1, indicating no influential cases. The Durbin–Watson statistic (DW = 1.982) fell within the acceptable range, consistent with the absence of residual autocorrelation. All continuous variables were standardized (*Z*-scores) prior to entry into the regression model. Ordinary Least Squares (OLS) regression was employed to evaluate the model, with results presented in Table 3. The linear regression analysis showed that, after controlling for nine covariates including gender, age and so on, traditional Chinese health-preserving exercises were significantly and positively associated with self-worth among older adults (*β* = 0.104, *t* = 2.227, *p* = 0.026 < 0.05). The regression model was statistically significant, *F*(10, 489) = 5.113, *p* < 0.001, *R*^2^ = 0.095, adjusted *R*^2^ = 0.076. These results indicated that traditional Chinese health-preserving exercises were positively associated with higher self-worth among older adults, providing support for Hypothesis 1. Although this association was statistically significant, the magnitude was small, with a standardized coefficient of 0.104 and only 9.5% of the variance explained (*R*^2^ = 0.095). In addition, the lower bound of the Bootstrap 95% confidence interval [0.013, 0.202] was close to zero, suggesting some uncertainty in the estimate.

**Table 3:** Regression of traditional Chinese health-preserving exercises on self-worth (*N* = 500).

	Self-worth
Constant	2.411^**^ (5.603)
TCHPE	0.104^*^(2.227)
Gender	−0.004(−0.099)
Age	0.111^*^ (2.120)
Practice format	0.183^**^(4.002)
Education background	−0.025(−0.417)
Economic income	0.117^*^(2.127)
Marital status	−0.028(−0.588)
Number of children	−0.085 (−1.738)
Living arrangement	0.043(0.988)
Physical condition	0.114^*^ (2.537)
*R^2^*	0.095
Adjusted *R*^2^	0.076
*F*	*F* (10,489) = 5.113, *p* = 0.000
D–W	1.982

Significance level: ^*^p < 0.05, ^**^p < 0.01, *t*-values in parentheses. TCHPE, traditional Chinese health-preserving exercises.

Among the control variables, gender (*β* = −0.004, *t* = −0.099), education background (*β* = −0.025, *t* = −0.417), marital status (*β* = −0.028, *t* = −0.588), number of children (*β* = −0.085, *t* = −1.738), and living arrangement (*β* = 0.043, *t* = 0.988) were not significantly associated with self-worth. By contrast, age (*β* = 0.111, *t* = 2.120, *p* < 0.05), practice format (*β* = 0.183, *t* = 4.002, *p* < 0.01), economic income (*β* = 0.117, *t* = 2.127, *p* < 0.05), and physical condition (*β* = 0.114, *t* = 2.537, *p* < 0.05) were all significantly and positively associated with self-worth.

Although gender, education background, marital status, number of children, and living arrangement were included as control variables, they were not significantly associated with self-worth. Age, practice format, economic income, and physical condition were significantly associated with self-worth. However, the total association between traditional Chinese health-preserving exercises and self-worth remained significant after controlling for these covariates. These results underscore the unique role of traditional Chinese health-preserving exercises.

### 4.4 Test of the mediating role of self-identity

The mediating role of self-identity was tested using the SPSS PROCESS macro (Model 4) to examine the indirect pathway through which traditional Chinese health-preserving exercises are associated with self-worth. The analysis was conducted with 5,000 bootstrap samples and 95% bias-corrected confidence intervals. An indirect pathway was considered statistically significant if the confidence interval did not include zero. Detailed results of the mediation analysis are presented in Tables 4, 5.

**Table 4:** Mediating pathway of traditional Chinese health-preserving exercise on self-worth (*N* = 500).

Variables	Self-worth (total association)			Self-identity			Self-worth (direct association)		
	*β*	SE	*T*	*β*	SE	*T*	*β*	SE	*T*
Gender	−0.004	0.084	−0.099	0.022	0.075	0.514	−0.021	0.057	−0.706
Age	0.111	0.059	2.120^*^	0.133	0.053	2.539^*^	0.013	0.040	0.363
Practice format	0.183	0.088	4.002^**^	0.175	0.078	3.851^**^	0.053	0.060	1.693
Education background	−0.025	0.042	−0.417	−0.018	0.037	−0.301	−0.012	0.028	−0.289
Living arrangement	0.043	0.114	0.988	0.088	0.102	2.032^*^	−0.022	0.077	−0.753
Physical condition	0.114	0.070	2.537^*^	0.065	0.063	1.457	0.066	0.047	2.158^*^
Economic income	0.117	0.051	2.127^*^	0.064	0.045	1.159	0.070	0.034	1.879
Number of children	−0.085	0.077	−1.738	−0.036	0.069	−0.732	−0.059	0.052	−1.772
Marital status	−0.028	0.091	−0.588	−0.008	0.081	−0.168	−0.022	0.061	−0.686
TCHPE	0.104	0.048	2.227^*^	0.124	0.043	2.680^**^	0.012	0.033	0.367
Self-identity							0.825	0.034	24.134^**^
*R* ^2^	0.095			0.101			0.587		
Adjusted *R*^2^	0.076			0.083			0.578		
*F*	5.113^**^			5.488^**^			63.122^**^		

Significance level: ^*^p < 0.05, ^**^p < 0.01. TCHPE, traditional Chinese health-preserving exercises.

**Table 5:** Bootstrap analysis of the mediation pathway.

Path	Value	Boot SE	Bootstrap 95% CI		*p*
			Boot LLCI	Boot ULCI	
Total association	0.104^*^	0.048	0.013	0.202	0.026
Direct association	0.012	0.033	−0.052	0.077	0.714
Indirect pathway	0.095^*^	0.038	0.018	0.165	0.013

Significance level: ^*^p < 0.05.

As shown in Table 4, after controlling for gender, age, practice format, education background, economic income, marital status, number of children, living arrangement, and physical condition, the total association between traditional Chinese health-preserving exercises and self-worth was significantly positive (*β* = 0.104, *t* = 2.227, *p* < 0.05). Traditional Chinese health-preserving exercises were significantly and positively associated with self-identity (*β* = 0.124, *t* = 2.680, *p* < 0.01), and self-identity was significantly and positively associated with self-worth (*β* = 0.825, *t* = 24.134, *p* < 0.01). However, the direct association between traditional Chinese health-preserving exercises and self-worth was not statistically significant (*β* = 0.012, *t* = 0.367, *p* > 0.05). Accordingly, Bootstrap 95% confidence intervals were used to further verify the mediating role of self-identity.

As shown in Table 5, the total association between traditional Chinese health-preserving exercises and self-worth was statistically significant [*β* = 0.104, *p* = 0.026 < 0.05, Bootstrap 95% CI (0.013, 0.202)]. With the inclusion of self-identity, the direct association was 0.012 [*p* = 0.714 > 0.05, Bootstrap 95% CI (−0.052, 0.077)]. As this confidence interval included zero, the direct association was not significant. These results indicated that self-identity explained a substantial portion of the association between traditional Chinese health-preserving exercises and self-worth, providing support for Hypothesis 2. Specifically, the indirect pathway was as follows: traditional Chinese health-preserving exercises → self-identity → self-worth. The coefficient for this indirect pathway was 0.095 [*p* = 0.013 < 0.05, Bootstrap 95% CI (0.018, 0.165)]. As this confidence interval did not include zero, the mediating pathway was statistically significant. Although this indirect pathway was statistically significant, the magnitude was small (0.095), and the lower bound of its Bootstrap 95% confidence interval [0.018, 0.165] was close to zero, suggesting some uncertainty in the estimate.

### 4.5 Test of the moderated mediation model

This study employed the SPSS PROCESS macro (Model 7) to test the moderating role of social support on the first-stage path of the mediation model (traditional Chinese health-preserving exercises → self-identity). A bias-corrected percentile bootstrap method with 5,000 resamples was used to estimate 95% confidence intervals. The moderation effect was examined by testing the significance of the interaction term and the conditional simple slopes. The moderated mediation effect was assessed via the index of moderated mediation, and was considered significant when the 95% confidence interval of this index did not include zero.

All interaction terms were computed from standardized variables. Because standardization sets each variable’s mean to zero, the resulting product terms were inherently mean-centered, eliminating the need for an additional centering step. As shown in Table 6, after controlling for gender, age, practice format, education background, economic income, marital status, number of children, living arrangement, and physical condition, the interaction between traditional Chinese health-preserving exercises and social support was significantly and positively associated with self-identity (*β* = 0.241, t = 3.692, *p* < 0.001). This finding indicates that social support positively moderated the first-stage path of the mediation model, that is, the association between traditional Chinese health-preserving exercises and self-identity.

**Table 6:** Moderated mediation model results (*N* = 500).

Variables	Self-worth			Self-identity		
	*β*	SE	*T*	*β*	SE	*T*
TCHPE	0.012	0.033	0.367	0.124	0.043	2.680^**^
Self-identity	0.825	0.034	24.134^**^			
Social support				0.106	0.205	0.518
TCHPE * social support				0.241	0.065	3.692^**^
Gender	−0.040	0.057	−0.706	0.103	0.069	1.491
Age	0.015	0.040	0.363	0.086	0.049	1.765
Practice format	0.102	0.060	1.693	0.219	0.072	3.049^**^
Education background	−0.008	0.028	−0.289	−0.016	0.034	−0.459
Living arrangement	−0.058	0.077	−0.753	0.081	0.093	0.871
Physical condition	0.102	0.047	2.158^*^	0.030	0.058	0.522
Economic income	0.064	0.034	1.879	0.013	0.041	0.321
Number of children	−0.092	0.052	−1.772	−0.007	0.063	−0.117
Marital status	−0.042	0.061	−0.686	0.045	0.074	0.614
*R* ^2^	0.587			0.259		
Adjusted *R*^2^	0.577			0.239		
*F*	63.122^**^			14.200^**^		

Significance level: ^*^p < 0.05, ^**^p < 0.01. TCHPE, traditional Chinese health-preserving exercises.

To further test H3, a simple slope analysis was conducted. As shown in Table 7, the association between traditional Chinese health-preserving exercises and self-identity varied across levels of social support. At low levels of social support, this association was negative and nonsignificant (*β* = −0.117, SE = 0.069, *t* = −1.699, *p* = 0.090); at medium levels, it was positive and significant (*β* = 0.124, SE = 0.043, *t* = 2.891, *p* = 0.004); and at high levels, it was strongest and significant (*β* = 0.365, SE = 0.057, *t* = 6.404, *p* < 0.001). These results indicate that the higher the level of social support, the stronger the positive association between traditional Chinese health-preserving exercises and self-identity, providing support for Hypothesis 3.

**Table 7:** Conditional simple slopes analysis results.

Social support level	*β*	SE	*t*	*p*
Low-level (−1 SD)	−0.117	0.069	−1.699	0.090
Medium-level (Mean)	0.124	0.043	2.891	0.004
High-level (+1 SD)	0.365	0.057	6.404	<0.001

A moderated mediation analysis was further conducted to examine whether the mediating role of self-identity varied across levels of social support, with the results of the conditional indirect pathway analysis presented in Table 8. Based on the mean ± 1 SD split, social support was categorized into low, medium, and high levels. At low levels of social support, the indirect pathway was not significant [*β* = −0.082, Bootstrap 95% CI (−0.196, 0.021)], as the confidence interval included zero. At medium levels of social support, the indirect pathway was likewise not significant [*β* = 0.031, Bootstrap 95% CI (−0.046, 0.100)], as the confidence interval included zero. However, at high levels of social support, the indirect pathway was significant [*β* = 0.143, Bootstrap 95% CI (0.052, 0.231)], as the confidence interval did not include zero. These findings indicated that high levels of social support moderated the mediating role of self-identity. Among older adults who received sufficiently high levels of social support, traditional Chinese health-preserving exercises were associated with greater self-identity, which involved affirmation from both the self and others, and this greater self-identity was in turn associated with higher self-worth.

**Table 8:** Conditional indirect pathway results.

Mediating variable	Level	Value	Boot SE	Boot LLCI	Boot ULCI
Self-identity	Low-level (−1SD)	−0.082	0.056	−0.196	0.021
	Medium-level (Mean)	0.031	0.037	−0.046	0.100
	High-level (+1SD)	0.143	0.046	0.052	0.231

Finally, to further test whether moderated mediation was established, the index of moderated mediation was reported. As shown in Table 9, this index was 0.199 [Bootstrap SE = 0.062, 95% CI (0.086, 0.330)], and its confidence interval did not include zero, indicating that moderated mediation was established. In terms of magnitude, the standardized coefficient of the interaction term was 0.241, of small-to-medium magnitude. The index of moderated mediation was 0.199, and its Bootstrap 95% confidence interval [0.086, 0.330], although entirely above zero, was relatively wide, suggesting some uncertainty in the estimate. By contrast, the conditional indirect pathway at high levels of social support was 0.143 [95% CI (0.052, 0.231)], with a relatively narrow confidence interval, indicating a more robust estimate.

**Table 9:** Index of moderated mediation.

Index of moderated mediation	Boot SE	Boot LLCI	Boot ULCI
0.199	0.062	0.086	0.330

### 4.6 Robustness checks

The sample for this study was drawn from 12 Tai Chi and Health Qigong associations, with 40–42 participants in each association (M = 41.7) and a total sample size of 500, such that the observations were nested within associations. To evaluate the potential clustering effect associated with this nested structure, we computed the intraclass correlation coefficients (ICCs) for the key variables. The ICCs for self-identity, social support, self-worth, and traditional Chinese health-preserving exercise were 0.018, 0.020, 0.030, and 0.000, respectively, all well below the commonly used threshold of 0.05, these values suggest that only a very small proportion of variance was attributable to the association level. Taking self-worth, the variable with the highest ICC (0.030), as the reference, the design effect [= 1 + (average cluster size − 1) × ICC] was approximately 2.2, corresponding to an effective sample size of approximately 225. This suggests that the sample remained sufficiently large even after the clustering structure was taken into account.

To further examine whether the key results were sensitive to clustering, we re-estimated the regression model for the interaction term using cluster-robust standard errors, with association specified as the clustering variable. The results showed that the interaction coefficient was unchanged (*β* = 0.241) and remained statistically significant after the cluster-robust correction (*p* < 0.001), with no change in the substantive conclusions. However, given that the analysis involved only 12 associations and no small-sample correction or multilevel model was applied, statistical inference based on cluster-robust standard errors may itself be unreliable; therefore, this robustness check should be viewed strictly as exploratory sensitivity evidence rather than as a definitive conclusion that clustering is inconsequential. It should also be noted that the mediation and moderated mediation analyses in this study (i.e., the indirect paths, the index of moderated mediation, and the conditional effects) were conducted using the PROCESS macro, which does not incorporate the clustering structure; accordingly, the primary PROCESS estimates and the indirect effects remain unadjusted for clustering, and the clustering structure was evaluated only in the supplementary regression described above. Nevertheless, although the ICCs of all four variables were extremely low (the highest being only 0.030) and the design effect was only 2.2, these low ICCs do not eliminate the possibility of unreliable variance estimation with so few clusters, and the robustness analysis cannot definitively establish the validity of individual-level inference.

Furthermore, given that marital status is inherently an unordered categorical variable, treating it as an ordinal score may artificially impose a linear ordering and equal spacing across its categories. To examine whether the study conclusions were sensitive to this coding approach, we recoded marital status into dummy variables (with married as the reference category, creating three dummy variables for unmarried, divorced, and widowed) and re-estimated the model (*N* = 500) while keeping all other variables and the model specification completely unchanged. The results showed that when marital status was treated as an unordered categorical variable (dummy coding), the interaction term remained statistically significant (*β* = 0.225, *t* = 4.80, *p* < 0.001), and the index of moderated mediation also remained significant [index = 0.166, bootstrap 95% CI (0.087, 0.259)]. Compared with the main analysis (ordinal coding, interaction *β* = 0.241, index = 0.199), the coefficients differed slightly, but the conclusions regarding significance were consistent. Regarding the conditional indirect pathways, at low levels of social support, the indirect pathway was significant in the dummy-coded model [−0.188, bootstrap 95% CI (−0.328, −0.059)], whereas it was not significant in the main analysis [−0.082, bootstrap 95% CI (−0.196, 0.021)]; this modest difference likely reflects the more appropriate treatment of marital status as an unordered categorical variable under dummy coding, which avoids the artificial linear ordering imposed by the ordinal score. At moderate levels, the indirect pathway was not significant in either model [−0.022, bootstrap 95% CI (−0.096, 0.048) vs. 0.031, bootstrap 95% CI (−0.046, 0.100)]. At higher levels, it remained significant in both models [0.145, bootstrap 95% CI (0.072, 0.226) vs. 0.143, bootstrap 95% CI (0.052, 0.231)]. The higher-level pathway was stable across the two coding schemes, with nearly identical estimates and confidence intervals that both lay well away from zero. In contrast, at low levels, the pathway was already marginally nonsignificant in the main analysis (with an upper confidence limit of 0.021 close to zero), and under dummy coding it was more clearly negative and significant, rather than reversing direction. These findings indicate that the core conclusions were robust to the coding of marital status.

## 5 Discussion and practical implications

### 5.1 The association between traditional Chinese health-preserving exercises and self-worth

The present finding indicated that participation in traditional Chinese health-preserving exercises was significantly associated with higher self-worth among older adults, supporting Hypothesis 1. Self-worth theory mainly concerns individuals’ cognition and evaluation of their personal value, particularly when facing challenges or pursuing goals (Covington, 2009). Traditional Chinese health-preserving exercises, such as Tai Chi, Baduanjin, and Wuqinxi, integrate movement, breath, and mental focus, and have been associated with better balance, flexibility, and cardiorespiratory function, as well as with reduced anxiety, depression, and loneliness, a pattern consistent with psychological well-being (Chair et al., 2025).

Through this integrated practice, older adults may experience a regained sense of bodily control. This renewed sense of mastery is associated with reduced helplessness accompanying physical decline and may be linked to rebuilding the foundation for self-worth. Socially, the collective nature of the practice is linked to the formation of a non-competitive support network, and most participants in this study preferred group practice. In this setting, older adults often become peer mentors and health advocates through experience sharing. Such role transitions offer opportunities for expressing self-worth and receiving social recognition. Moreover, rooted in philosophies of “human-nature harmony,” “mind–body unity,” and “holistic balance,” the practice itself represents a form of perception and introspection regarding the laws of life. This process relates to a shift in focus from external achievements toward inner peace and the meaning of life, a pathway associated with gaining both recognition of and satisfaction with self-worth (Blake and Hawley, 2012).

Drawing on the patterns of association identified above, we offer several preliminary practical implications. First, at the level of exercise type and intensity, the present study found that the association between traditional Chinese health-preserving exercises and self-worth remained robust (*β* = 0.104) even after accounting for physical condition. This suggests that the relevance of such exercises to self-worth does not appear to vary substantially with individual differences in health status. Traditional Chinese health-preserving exercises are characterized by slow movements, controllable intensity, and low physical load, features that may be broadly applicable across diverse health levels. These findings point to the potential value of moderate- to low-intensity activities such as Tai Chi or Baduanjin for older adults; the specific frequency, duration, and intensity best suited to self-worth, however, remain to be examined in future research. Second, at the level of mind–body awareness, traditional Chinese health-preserving exercises integrate movement, breath, and intention in a coordinated manner, offering older adults a pathway through which sustained bodily perception is associated with a renewed sense of self-mastery and reduced feelings of powerlessness that often accompany age-related physical decline. Mindfulness guidance, for instance a brief breathing-awareness practice at the start of group instruction, may be associated with a shift in older adults’ attentional focus from “physical decline” to “physical efficacy.” Through each deliberate movement, they may gradually accumulate embodied competence experiences, a process that may accompany the development of self-worth. Third, at the level of individual adaptation, the present study found that age and physical condition were significant covariates of self-worth, suggesting that the suitability of exercise-related implications may differ across older adults of varying ages and health statuses. These findings imply that the content and intensity of exercise may need to be adapted to individual age, health status, and physical function, a consideration relevant to both safety and sustainability.

### 5.2 The mediating role of self-identity

The present finding demonstrated that participation in traditional Chinese health-preserving exercises was significantly associated with higher self-worth among older adults, and that this association operated primarily through the indirect pathway of self-identity, supporting Hypothesis 2. Self-identity theory conceptualizes identity as arising from the dynamic interplay between individual construction and social shaping, reflecting the interaction between subjectivity and sociality (Horowitz, 2012). Older adults engage in traditional Chinese health-preserving exercises as a medium through which the development of self-identity may be supported across physiological, psychological, and social dimensions.

Physiologically, the standardized movements and therapeutic properties of these exercises allow older adults to regain a sense of bodily control, thereby reframing the aging body from a site of inevitable decline to a system capable of self-regulation. This transformation may constitute the foundation for rebuilding a positive bodily identity (Park et al., 2023). Psychologically and socially, the social networks formed through group practice are associated with a greater sense of belonging and cohesion among older adults. Within this context, older adults may assume new social roles, responsibilities, and a sense of purpose, a process that may be interpreted as reflecting a transformation of their self-identity from that of “social burden” to “social strength.” This mediating pathway may be associated with a shift from a negative to a positive self-identity, which in turn may represent a key psychological pathway linked to higher overall self-worth (Polacsek et al., 2022).

Drawing on the mediation patterns observed in this study, we offer several preliminary practical implications. First, at the level of instructional content, the present findings indicate that the indirect pathway through self-identity explained a substantial portion of the association between traditional Chinese health-preserving exercises and self-worth, suggesting that the positive correlates of exercise may be linked to identity-related processes rather than to direct physiological pathways alone. In light of this, exercise programs might incorporate health-preserving theory and philosophical principles alongside physical training, rather than focusing exclusively on motor skill acquisition. Introducing older adults to the cultural concept of “harmony between humans and nature” during movement practice may provide a cognitive basis relevant to the development of self-identity. Second, at the level of role development, the findings point to a pattern in which older adults engaged in collective practice showed higher self-identity, which may be interpreted as reflecting a shift in self-perception from “social burden” to “social contributor,” a transition that appears central to identity development. Establishing senior peer instructor roles within group settings and inviting experienced practitioners to serve as mentors may be associated with the consolidation of this shift, as the act of guiding fellow practitioners offers ongoing opportunities to enact and internalize a newly acquired positive identity. Third, at the level of the practice environment, traditional Chinese health-preserving exercises are rooted in the philosophy of harmony between humans and nature, and the practice itself carries an inherently introspective quality. It is an engagement with the rhythms of life through bodily experience in natural settings. Prioritizing locations such as parks, lakesides, and Tai Chi plazas for regular practice may be associated with a deepening of this embodied experience, grounding identity-related change in authentic, situated interaction between the body and the natural world.

### 5.3 The moderating role of social support

The moderated mediation analysis revealed that, compared with low or moderate levels of social support, the indirect association between traditional Chinese health-preserving exercises and self-worth through self-identity was significant only under high levels of social support. Specifically, social support moderated the first-stage path of the mediation, that is, the association between traditional

Chinese health-preserving exercises and self-identity, confirming Hypothesis 3. According to social support theory, a stronger social support network could enhance individuals’ capacity to cope with various environmental challenges (Sarason and Sarason, 2013). Under high levels of social support, including emotional support from family, encouragement from peers, and recognition from the community, these sources of support may serve as tangible forces through which older adults’ self-worth is affirmed.

On the one hand, such support is associated with reduced frustration arising from unfamiliarity with movements during the early stage of exercise, is linked to sustained motivation to participate, and offers a foundation for the positive transformation of self-identity (Steinhoff and Reiner, 2024). On the other hand, through positive feedback, role validation, and the cultivation of a collective identity within the group, it is associated with strengthened internalization of the new identity acquired through exercise, a process linked to higher self-worth (Zimmer et al., 2023). This result corroborated the findings of Sheehy et al. (2016). Conversely, under low levels of social support, the transformation of self-identity through participation in traditional Chinese health-preserving exercises may be constrained by insufficient support from family, peers, and the community. Under such conditions, the indirect association may be attenuated, and older adults’ self-worth tends to be lower.

Drawing on the patterns identified above, we outline several preliminary practical implications structured across three interconnected dimensions. First, at the level of program design, the present study found that the conditional indirect pathway reached statistical significance exclusively under high levels of social support, with neither low nor moderate levels reaching significance. This pattern suggests that social support may play a central rather than a supplementary role in the psychological benefits of exercise: in the absence of adequate social support, the mediating pathway may not operate. These findings point to the potential value of structured group-based formats that embed interactive elements into the exercise curriculum, such as partnered practice routines, achievement-sharing sessions, and reflective group discussions. Such formats may be associated with a more supportive social milieu, particularly for older adults whose existing social support is limited. Second, at the level of institutions, the present study identified positive feedback and role affirmation from the broader community as key sources through which social support operates as a moderator. Local communities may provide designated venues for group exercise activities and offer institutional recognition, such as program completion certificates or integration into community health services. This form of institutionally anchored support may be particularly relevant for older adults whose personal social support networks are limited. Third, at the level of the activity ecosystem, community-based group exercise may be linked with health education lectures, cultural showcases, and intergenerational exchange programs, potentially broadening the scope of older adults’ social participation. This integration may extend the sources of social support from within a single exercise group to a more expansive social network, which may be linked to their self-identity and sense of self-worth.

### 5.4 Limitations and future directions

First, cross-sectional design and sample generalizability. The sample comprised long-term practitioners selected from 12 Tai Chi and Health Qigong associations, all of whom engaged in organized, sustained practice. Given the cross-sectional design, the findings reflect patterns of association among variables rather than causal pathways. A mediation model with bootstrapping was therefore employed to delineate the relational structure among the variables without asserting causal direction, yielding clear and testable hypotheses to guide future longitudinal inquiry. Accordingly, the conclusions carry strong relevance for older adults who participate in organized, long-term practice, and the extent to which these findings extend to non-organized or infrequently practicing older populations remains an open empirical question. Future research could draw on broader community samples, include a non-practitioner control group, and adopt a longitudinal tracking design to establish causal inference, thereby testing and expanding the boundary conditions of the pathways documented here. The present findings also offer a preliminary empirical foundation for subsequent longitudinal and causal verification work.

Second, generalizability of the moderated mediation model. The moderated mediation model tested in this study was significant only under high social support, suggesting that the indirect pathway from traditional Chinese health-preserving exercises to self-worth through self-identity does not generalize uniformly to all older adults. This pattern represents both a theoretical finding and a boundary condition of the present study. For older adults with limited social support, exercise participation alone may not be associated with higher self-worth through the self-identity pathway. Future research can address this limitation in two ways. On the one hand, an intervention combining traditional Chinese health-preserving exercises with social support enhancement could be designed to examine whether embedding peer support or group interaction within exercise programs extends this indirect pathway to older adults with low or medium social support. On the other hand, the moderated mediation model should be retested in larger and more representative samples to further verify the pattern of indirect pathways across different levels of social support.

Third, future qualitative research is warranted. Although the cross-sectional quantitative design reveals patterns of association among variables, it cannot fully capture the subjective experiences and psychological changes older adults undergo while participating in traditional Chinese health-preserving exercises. Future research could adopt qualitative methods such as semi-structured interviews to gain deeper insight into older adults’ motivations for engaging in such exercise, their sustained experiences, and the specific pathways through which they derive a sense of self-worth. Such inquiry would complement the quantitative approach by providing process-level understanding and further enriching knowledge of the pathways linking traditional Chinese health-preserving exercise to self-worth in older adults.

Fourth, nested data structure and cluster-based inference. The sample was nested within 12 associations, and although the intraclass correlation coefficients (ICCs) of the four variables were all extremely low (the highest being only 0.030), suggesting that clustering has a limited bearing on the results, cluster-robust inference with only 12 clusters may itself be unreliable. This should be taken into account when interpreting the robustness check results. Future research could adopt a non-nested sampling design, increase the number of associations, or employ multilevel models to further examine the role of the clustering structure.

Fifth, measurement consideration. The average variance extracted (AVE) for the social support scale was 0.455, slightly below the conventional threshold of 0.50, suggesting that the convergent validity of this measure is limited. Nevertheless, the composite reliability (CR = 0.769) and model fit of this scale were satisfactory, indicating adequate internal consistency. Therefore, the social support construct should be interpreted with some caution. Future research could further examine the psychometric properties of this scale in Chinese older adult populations, or employ an instrument with stronger convergent validity to verify the findings.

## 6 Conclusion

Grounded in self-worth theory, self-identity theory, and social support theory, the present study examined the association between traditional Chinese health-preserving exercises and self-worth among older adults, together with the mediating pathway of self-identity and the moderating role of social support. The findings indicated a significant positive association between traditional Chinese health-preserving exercises and older adults’ self-worth, and this association may be partially explained by the indirect pathway of self-identity. Furthermore, social support moderated the association between traditional Chinese health-preserving exercises and self-identity, such that this association was stronger at higher levels of social support, and the mediating role of self-identity was significant only at high levels of social support. Based on these findings, several preliminary practical implications may be considered. Older adults may engage in moderate- to low-intensity activities such as Tai Chi or Baduanjin; experienced practitioners may serve as peer instructors, a role that may be associated with the consolidation of their self-identity and self-worth; and structured group-based formats incorporating partnered practice routines, achievement-sharing sessions, and reflective group discussions may be linked to older adults’ connection with the broader community.

## Data Availability

The data analyzed in this study is subject to the following licenses/restrictions: the data that support the findings of this study are not openly available and are available from the corresponding author upon reasonable request. Requests to access these datasets should be directed to Huijun Han 800004@gzsport.edu.cn.
